# The Nearctic-Caribbean species *Leptotrachelus dorsalis* (Fabricius, 1801): Larval descriptions with a diagnosis of immature Ctenodactylini and natural history notes on the genus and tribe (Coleoptera, Carabidae)

**DOI:** 10.3897/zookeys.194.3308

**Published:** 2012-05-17

**Authors:** Terry L. Erwin, William H. White

**Affiliations:** 1 Hyper-diversity Group, Department of Entomology, MRC-187, National Museum of Natural History, Smithsonian Institution, Washington, P.O. Box 37012, DC 20013-7012, USA; 2 USDA, ARS, Sugarcane Research Laboratory, Houma, LA 70360, USA

**Keywords:** Sugarcane Savior Beetle, Louisiana, commensalism, Sugarcane, *Saccharum officinarum* L., Sugarcane Borer, *Diatraea saccharalis* (Fabricius)

## Abstract

Adults and larvae of *Leptotrachelus dorsalis* (Fabricius), the Sugarcane Savior Beetle, live in association with grasses, the larvae in the appressed leaf axils. Both adult and larval *Leptotrachelus dorsalis* eat larvae of the Sugarcane Borer, *Diatraea saccharalis* (Fabricius), and perhaps other insects living in the confines of the leaf sheaths of that and other grass-like species. The geographic range of *Leptotrachelus dorsalis* extends from Kansas in the west to the Atlantic seaboard, north as far as Ontario, Canada and south to Cuba; it is an eastern species of North America and the Caribbean. Larval character attributes that are shared with a related ctenodactyline, *Askalaphium depressum* (Bates), provide a preliminary basis for characterization of the immatures of tribe Ctenodactylini.

## Abstract (es)

**Resumen**

Los adultos y larvas de *Leptotrachelus dorsalis* (Fabricius), el Escarabajo Salvador de la Caña de Azúcar, viven asociados a hierbas, las larvas en las axilas de las hojas recostadas. Tanto el adulto como la larva de *Leptotrachelus dorsalis* se comen la larva del Taladrador de la Caña de Azúcar, *Diatraea saccharalis* (Fabricius), y quizás otros insectos más que viven en los confines de la vaina de la hoja de esta y otras especies de gramíneas. La distribución geográfica de *Leptotrachelus dorsalis* se extiende desde Kansas en el oeste hasta el litoral del Atlántico, hacia el norte hasta Ontario, Canadá, y hacia el sur hasta Cuba; es una especie del este de América del Norte y el Caribe. Los atributos de los caracteres larvales que son compartidos con un ctenodactylino, *Askalaphium depressum* (Bates), proveen una base preliminar para la caracterización de los inmaduros de la tribu Ctenodactylini.

## Introduction

**Figure 1.**
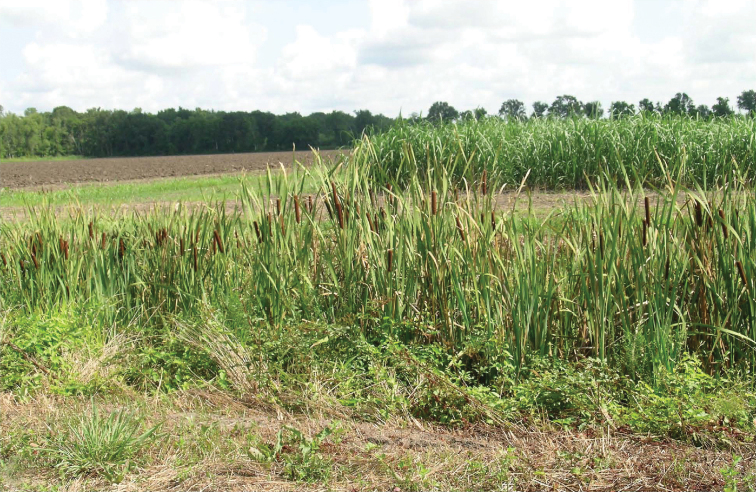
A stand of Cattails, *Typha latifolia* L. (foreground) near the edge of a sugarcane field (background) in the environs of Houma, LA. Insert: Photo credit: Randy Richard of the USDA, ARS Sugarcane Research Unit.

**Figure 2.**
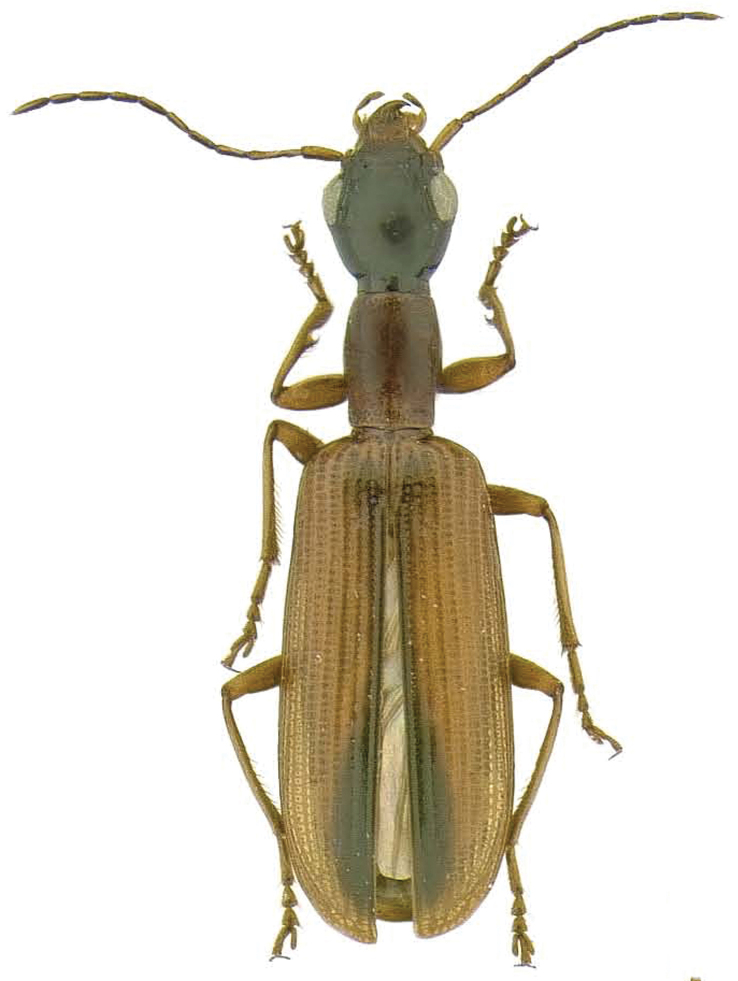
Adult, dorsal aspect, of *Leptotrachelus dorsalis* (Fabricius); adult from Wittman, Talbot County, MD. Apparent body length (ABL) = 8.1mm.

*Leptotrachelus dorsalis* (Fabricius) is known to occur in Canada – ON; Cuba; and the USA – AL, AR, CT, DC, DE, FL, GA, IA, IL, IN, KS, KY, LA, MD, MI, MN, MO, MS, NC, NJ, NY, OH, PA, SC, SD, TN, VA, and WV. According to Lindroth (1969), adults (Fig. 2) are found at the borders of pools and ponds where the vegetation is tall and rich, e.g., in cattail (*Typha latifolia* L.) swamps (Fig. 1) with tufts of *Carex rostrata* Stokes, *Menyanthes* sp., *Solanum dulcamara* L., and others. Adults take cover within the leaf axils of *Typha latifolia* stems. Adults are attracted to lights. In sugarcane, they are found within the canopy (top quarter) of the plant, the region of the sugarcane plant where the Sugarcane Borer, *Diatraea saccharalis* (Fabricius) lays its eggs and where the neonate larvae become established behind the leaf sheaths of elongating internodes. Larvae of *Leptotrachelus dorsalis* are rarely encountered below this region, as young Sugarcane Borer larvae are also rarely encountered in association with the lower mature internodes.

Erwin and Medina (2003) wrote: “Van Emden (1942) described the larva of *Leptotrachelus dorsalis* (Fabricius), Ctenodactylini, the only known larva of the tribe until now. However, at that time “Colliurina” and “Ctenodactylina” were regarded as subtribes of Colliurini (Colliurini is now classified as an unrelated clade, Odacanthini), thus van Emden inadvertently gave a single combined description for both tribes, and then provided in a key the means of which to separate what he regarded as subtribes, but with very few important features listed. Van Emden provided illustrations for many other genera in his important contribution to knowledge of carabid larvae, but did not provide any for these particular ctenodactyline and odacanthine groups.”

Now that we have reared specimens (all stages except egg), we realize that Van Emden’s description is not that of purely *Leptotrachelus dorsalis* individuals.

Thompson (1979) summarized van Emden’s description, but did not add anything new to it, did not provide illustrations of any Ctenodactylini, nor did he sort the mixed characters of the two tribes. He did provide illustrations (heads of L_1_ and L_3_ and cerci) of *Colliuris pensylvanicus* L. along with a reasonably complete description. These tribes, Ctenodactylini and Odacanthini, however, are now recognized as not being especially closely related (Erwin 1991).

Bionomics of *Leptotrachelus dorsalis* are discussed in a separate paper (White et al. in press). Here we provide larval and pupal descriptions of the Sugarcane Savior Beetle and notes on the taxonomic complementarity of *Leptotrachelus* and *Askalaphium* larvae as a beginning in understanding the immature forms of Ctenodactylini.

## Specimens and methods

Specimens were initially obtained from experiments to determine economic thresholds for Sugarcane Borer in new sugarcane cultivars (White et al. 2008).

Descriptive and larval preparation methods follow those suggested by the classic carabid larval method paper of Bousquet and Goulet (1984), particularly their coding system for setae and pores in L_1_ larvae, and their description format, believed to be ancestral in carabids. Additional setal and pore positions were discovered in Ctenodactylini (Erwin and Medina 2003) and were designated in a sequence that follows the methods suggested by Bousquet and Goulet (1984). However, we have not attempted that here since we do have the first instars. Those subsequent designations in Erwin and Medina (2003) may be merely accessory setae common in later instars. All *Leptotrachelus dorsalis* immature specimens illustrated here are from populations in the environs of Houma, LA. They were reared in the laboratory from eggs laid by wild captured adults in sugarcane plantings. Many additional larvae were hand collected in the leaf axils of standing sugarcane, as well.

### Tribe Ctenodactylini

(The following is based on larvae of *Askalaphium depressum* (Bates) and *Leptotrachelus dorsalis* (Fabricius), the only confirmed described larvae in the Tribe).

**Recognition.** (See Erwin and Medina 2003, and Fig. 14 herein for illustrations of *Askalaphium depressum*)Head and body depressed, markedly so in *Askalaphium depressum*, much less so in *Leptotrachelus dorsalis*. Head wider than prothorax in *Askalaphium depressum*, coequal in width in *Leptotrachelus dorsalis*. First instars of *Askalaphium depressum* unknown, in first instar of *Leptotrachelus dorsalis* frontal piece with long U-shaped row of short stiff setae likely used as an egg burster. Frontale slightly produced medially, toothed or shallowly bilobed. Neck slightly to moderately constricted, short and broad, cervical groove and keel distinct. Mandible with inner edge of blade and posterior margin of retinaculum moderately serrate (3^rd^ instar). Maxilla with inner lobe present, unisetose, seta 2× length of lobe in *Askalaphium depressum*, absent and devoid of seta in *Leptotrachelus dorsalis*. Labium markedly produced medially and unisetose. Antennomere 3 with small tubercule laterad near apex (not a hyaline bulb). Segment IX with two stout curved setae ventrally; urogomphi non-segmented, multi-nodose, infuscated. Pygopod with marked triangular patch of setae postero-ventrally.

### Key to the larvae of genera of Carabidae (in part)

(Modified from Thompson 1979)

**Table:** 

27 (25)	Blade of mandible and/orretinaculum denticulate or crenulate	28
–	Blade of mandible andretinaculum not denticulate or crenulate	36
28 (27)	Cervicalgroove present, short	29
–	Cervical groove absent	32
29 (28)	Antennae distinctly longer than mandibles	Panagaeini (in part)
–	Antennae subequal to mandibular length 30
30 (29)	Nasale medially produced, equally quatro-dentate; maxilla with inner lobe (L_3_); pygopod with dense patch of setae ventrally (L_3_); cerci not articulated	Ctenodactylini
–	Nasale not produced, margin medially microdentate, two lateral teeth larger than medial dents; maxilla without inner lobe (L_3_); pygopod without dense patch of setae ventrally (L_3_); cerci articulated	Odacanthini

### Key to the larvae of known genera of Ctenodactylini

**Table:** 

1	Head and body markedly depressed; head with definitive neck; tarsus multispinose, spines robust; pygopod multisetiferous medio-ventrally, setae curved, decumbent posteriorly	*Askalaphium* Liebke, 1938
–	Head and body slightly depressed; head without definitive neck; tarsus bisetose, setae fine; pygopod without medio-ventral patch of curved setae, general setae normal, straight, not decumbent	*Leptotrachelus* Latreille, 1829

#### Leptotrachelus dorsalis

(Fabricius, 1801)

http://species-id.net/wiki/Leptotrachelus_dorsalis

##### Description of first and third instars.

**Figure 3.**
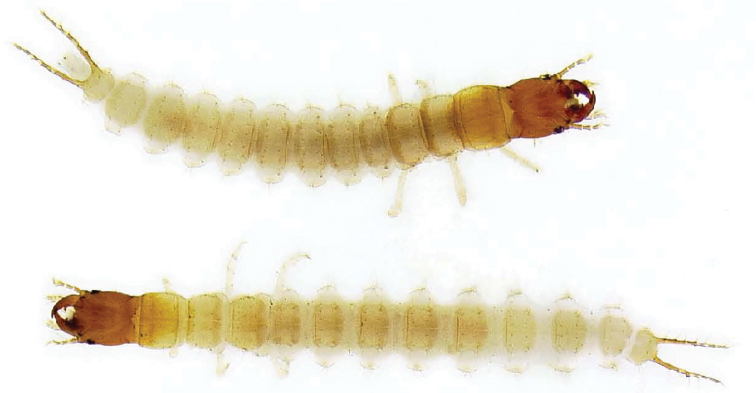
Larva, 3^rd^ instar (top), 2^nd^ instar (bottom), dorsal aspect, of *Leptotrachelus dorsalis* (Fabricius). Apparent body length (ABL) (L_3_ = 8.9mm; L_2_ = 7.0)

*Coloration* (as in Fig. 3). Mostly pale cream color with infuscated head capsule, mandibles, and urogomphi, the latter with pale spots; other mouthparts, antennae, and pronotum slightly darker than rest of body.

##### Microsculpture.

Head capsule without visible sculpticels.

##### Form.

**Figure 4.**
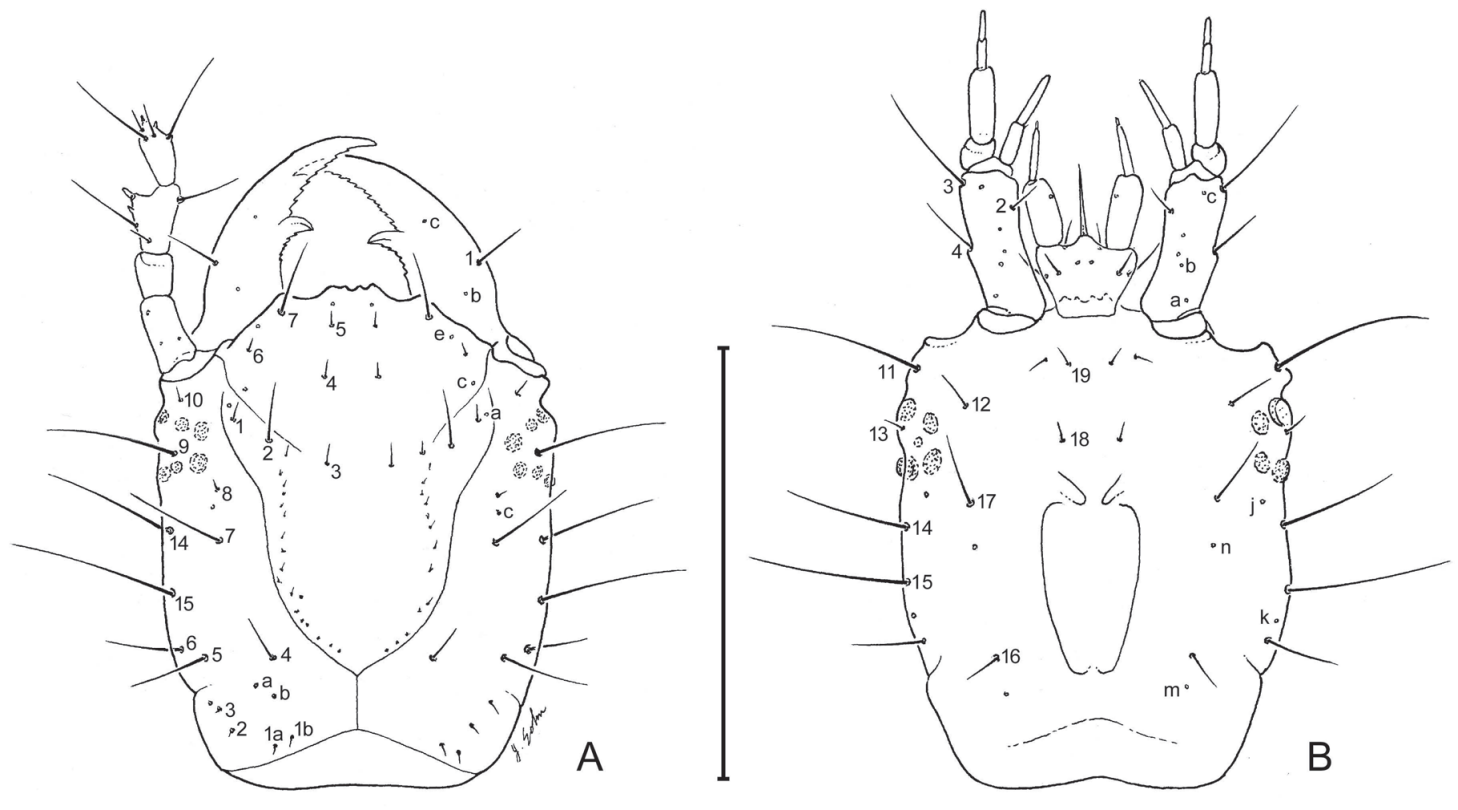
Larval head capsule (L_1_) of *Leptotrachelus dorsalis* (Fabricius). **A** dorsal aspect **B** ventral aspect. Scale line equals 0.5 mm.

**Head** (Figs 3, 4, 8). Nasale moderately produced, quarto-dentate, teeth coequal in length; mandibles robust and with obvious serrations medially on blade and posterior to retinaculum; genae not prominent, very slightly wider than distance across stemmata, slightly narrowed to broad neck. Eyes of 6 barely prominent stemmata. Antennomere slightly shorter than porrect mandible; antennomere 2 slightly shorter than 1, 3, and 4. Mandible with prominent retinaculum, curved dentiform; terebral blade obviously serrate, pensillus absent. Ligula of labium slightly produced, unisetose, labrum ventrally sextasetose. Ratios of palpomere lengths can be deduced from the illustrations.

##### Thorax.

**Figure 5.**
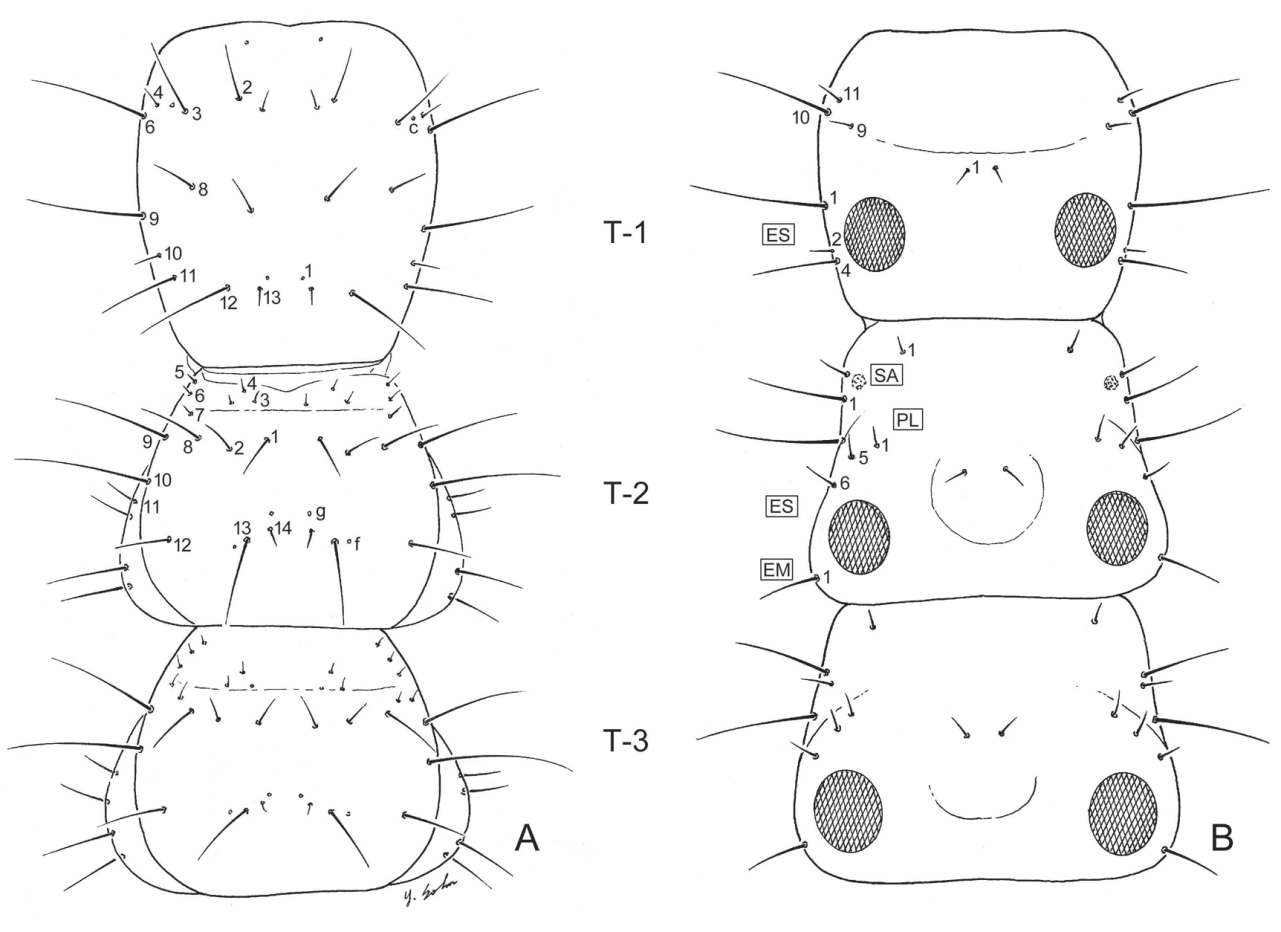
Larval thorax (L_1_), of *Leptotrachelus dorsalis* (Fabricius). **A** dorsal aspect **B** ventral aspect. Prothorax **T-1** Mesothorax **T-2** Metathorax **T-3** Episternum **ES** Epimeron **EM**. Scale line equals 0.5 mm.

(Figs 3, 5, 9). Prothorax narrowly quadrate (L_1_), more broadly quadrate (L_3_); meso- and metathorax transverse trapezoid, narrow anteriad, broader posteriorly.

##### Abdomen.

**Figure 6.**
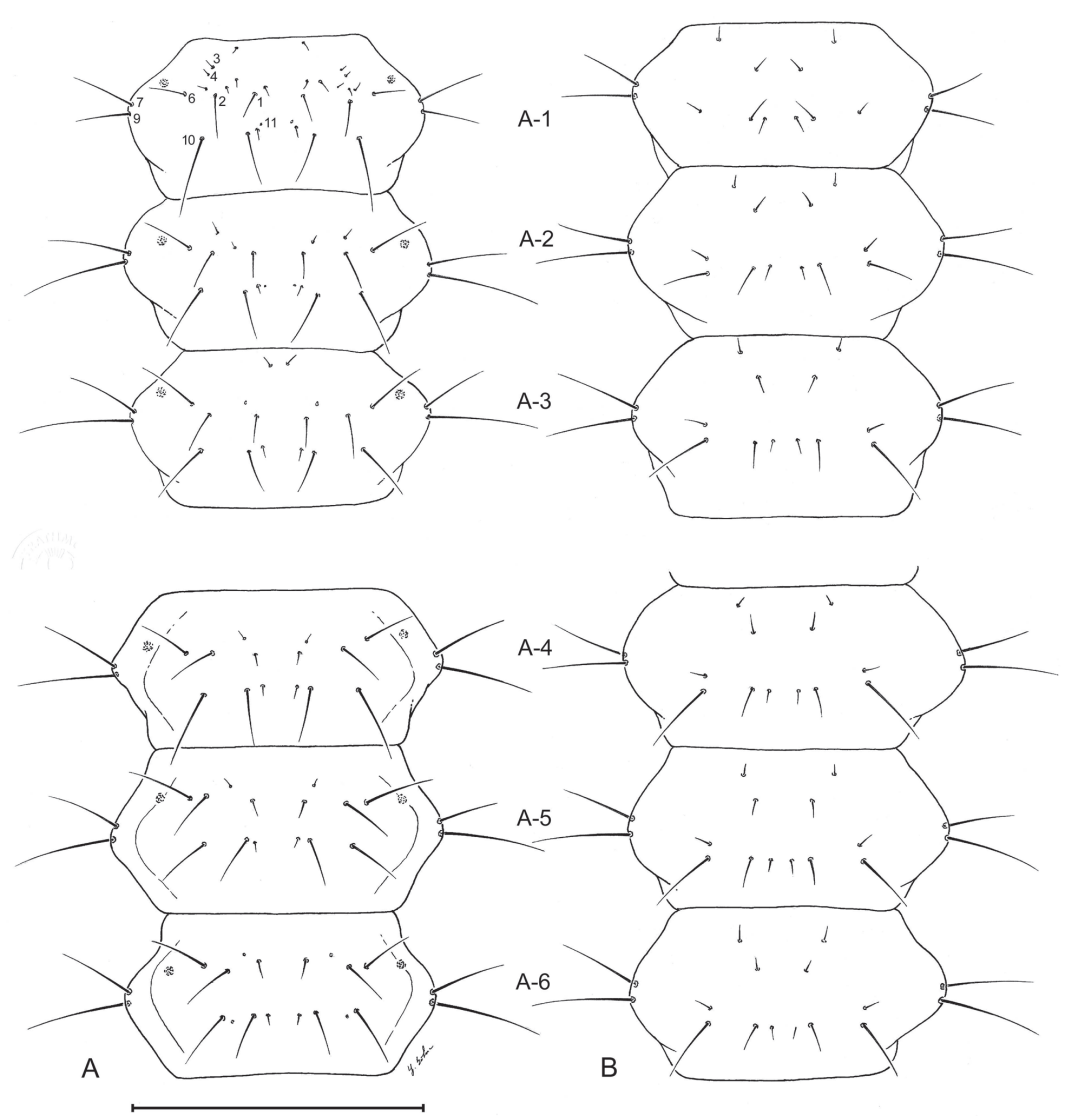
Larval abdomen (L_1_), of *Leptotrachelus dorsalis* (Fabricius). **A** dorsal aspect **B** ventral aspect. Abdominal segments **A-1** through **A-6**. Scale line equals 0.5 mm.

**Figure 7.**
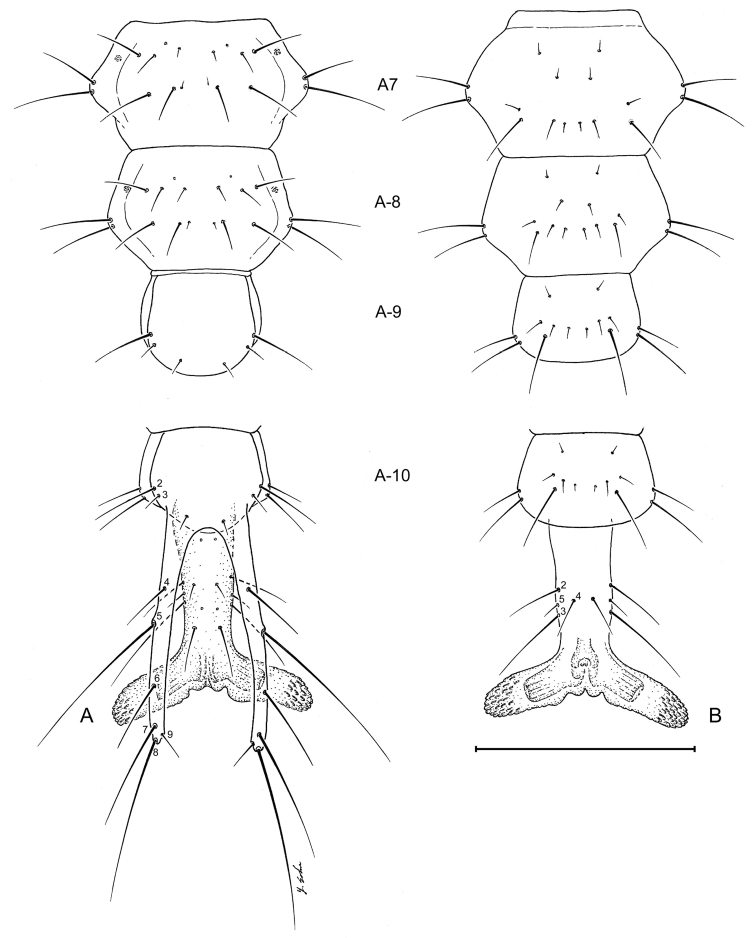
Larval abdomen (L_1_), of *Leptotrachelus dorsalis* (Fabricius). **A** dorsal aspect **B** ventral aspect. Abdominal segments **A-7** through **A-10**; and cerci and pygidium. Scale line equals 0.5 mm.

(Figs 3, 6, 7, 10, 11). Segments hexagonal, broad. Urogomphi about one and a half times as long as prothorax is long.

##### Legs.

(Figs 3, 12). Tarsus unispinose at apex and with a single seta at midpoint dorsally.

##### Chaetotaxy (L_1_).

**Head.** Frontale (Fig. 4) with 7 setae (FR1 **–** FR7) on each side; and 2 pores (FRc &FRe) on each side; egg burster a lyre-shaped row of short setae. Parietale (Figs 4A, 4B) with 19 setae (PA1 **–** PA19) and 5 pores (PAc, PAj, PAk, PAm, PAn) on each side. Antenna (Fig. 4A): antennomere 1 with 3 pores (unlabeled); antennomeres 2 **–** 4 with no pores; antennomere 3 with 3 setae (AN1 **–** AN3) and 1 small sensilla near base of sensorial appendage (Fig. 4A); antennomere 4 with 4 setae (AN1 **–** AN4) and 2 small apical sensillae (Fig. 4A). Mandible (Fig. 4A) with 1 seta (MN1) and 2 pores (MNb **–** MNc). Labium (Fig. 4B): prementum with 3 setae (LA2, LA3, LA7) and 1 pore (LAa) on each side; palpomere 1 with 1 pore (LAb); palpomere 2 and 3 without visible features. Maxilla (Fig. 4B): cardo without setae; stipes with 3 constant setae (MX2, MX3, MX4); 5 pores (MXa **–** MXc), others not labeled; lacinia and galeomeres without setae and pores; maxillary palpomeres without visible sensatory features.

##### Thorax.

Prothorax: Notum (Fig. 5A) with 10 major setae (PR2 **–** 4, 6, 8 **–** 13), PR1, 5, 7 absent, and 3 pores (PRc only named one) on each side; pleurite (Fig. 5B) with 3 setae (PL9, 10,11), and no pores on each side; episternum (Fig. 5B) with 3 setae (unnumbered).

Mesothorax: Notum (Fig. 5A) with 14 setae (ME1 **–** ME14), 3 small auxiliary setae, and 1 pores (MEg) on each side; episternum (Fig. 5B) with no setae and no pores; epimeron (Fig. 5B) with 1 seta (EM1); pleurite (Fig. 5B) with 3 posterior seta (PL1, 5, 6); sternum (Fig. 5B) with 1 seta (not numbered) and no pores on each side.

##### Abdomen.

Tergite I (Fig. 7A) with 10 setae (TE1 **–** TE10, TE8 missing and several accessory setae present) and 1 pore on each side. Tergites II **–** VIII as in Tergite 1 but with less accessory setae. Tergite IX and urogomphi (Fig. 7A) with 8 setae (UR1 **–** UR8, UR1 missing) and no pores. Epipleurite (Fig. 7B) with 2 setae (unnumbered) and no pores. Hypopleurite (Fig. 7B) with 7 setae (unnumbered) and no pores. Sterna 1 **–** 9 (Fig. 7B) with 5 or 6 setae each side (unnumbered) all in the same pattern. Sternum IX with 4 setae (ST2 **–** ST5) on each side.

**Figure 8.**
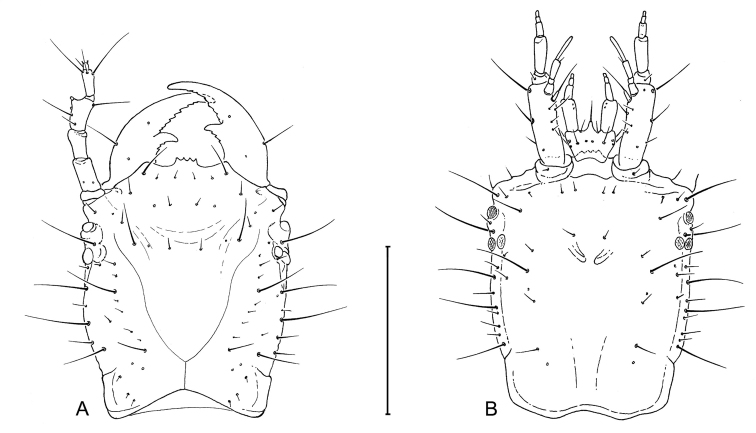
Larval head capsule (L_3_) of *Leptotrachelus dorsalis* (Fabricius). **A** dorsal aspect **B** ventral aspect. Scale line equals 0.5 mm.

**Figure 9.**
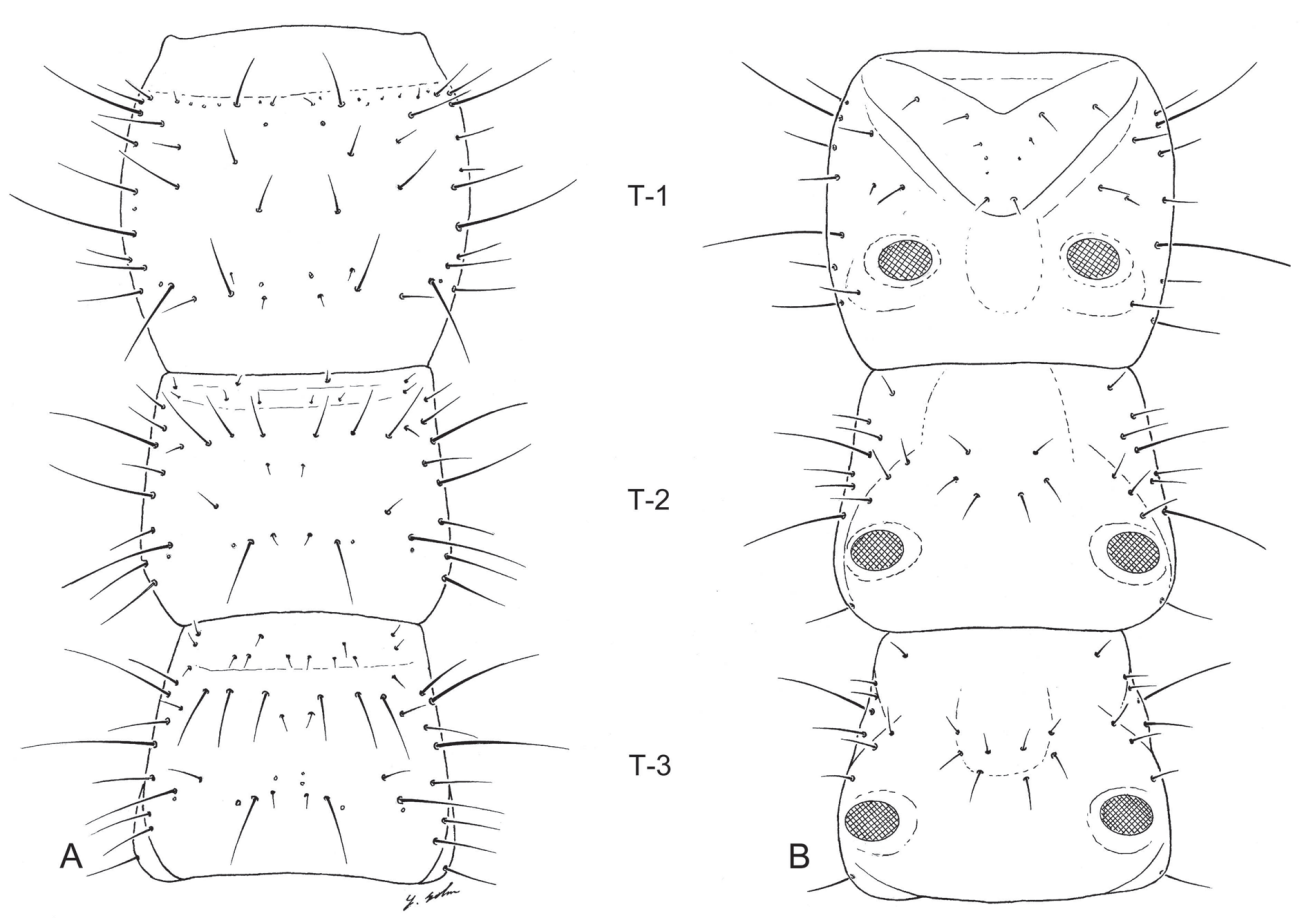
Larval thorax (L_3_), of *Leptotrachelus dorsalis* (Fabricius). **A** dorsal aspect **B** ventral aspect. Prothorax **T-1** Mesothorax **T-2** Metathorax **T-3**. Scale line equals 0.5 mm.

**Figure 10.**
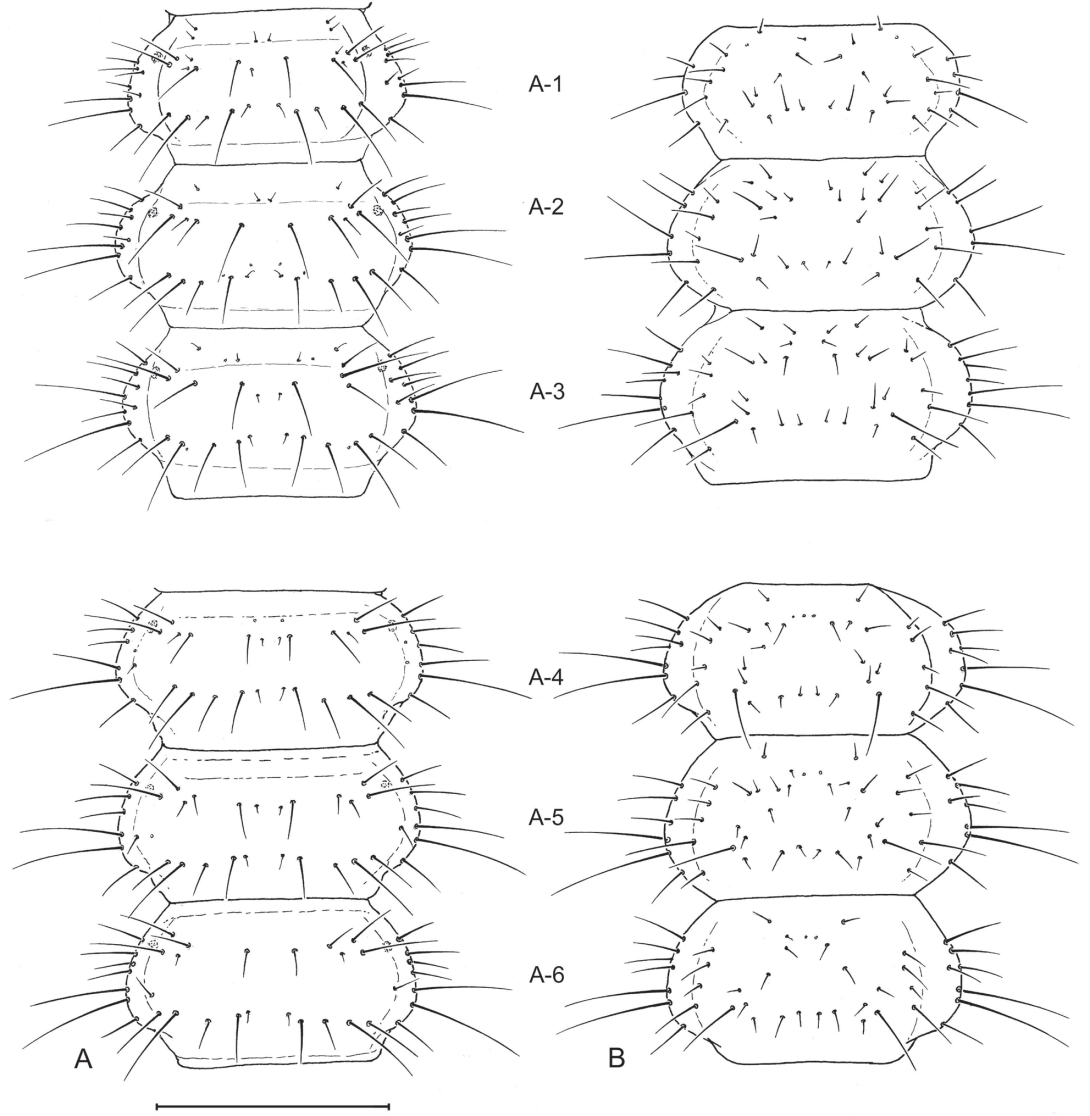
Larval abdomen (L_3_), of *Leptotrachelus dorsalis* (Fabricius). **A** dorsal aspect **B** ventral aspect. Abdominal segments **A-1** through **A-6**. Scale line equals 0.5 mm.

**Figure 11.**
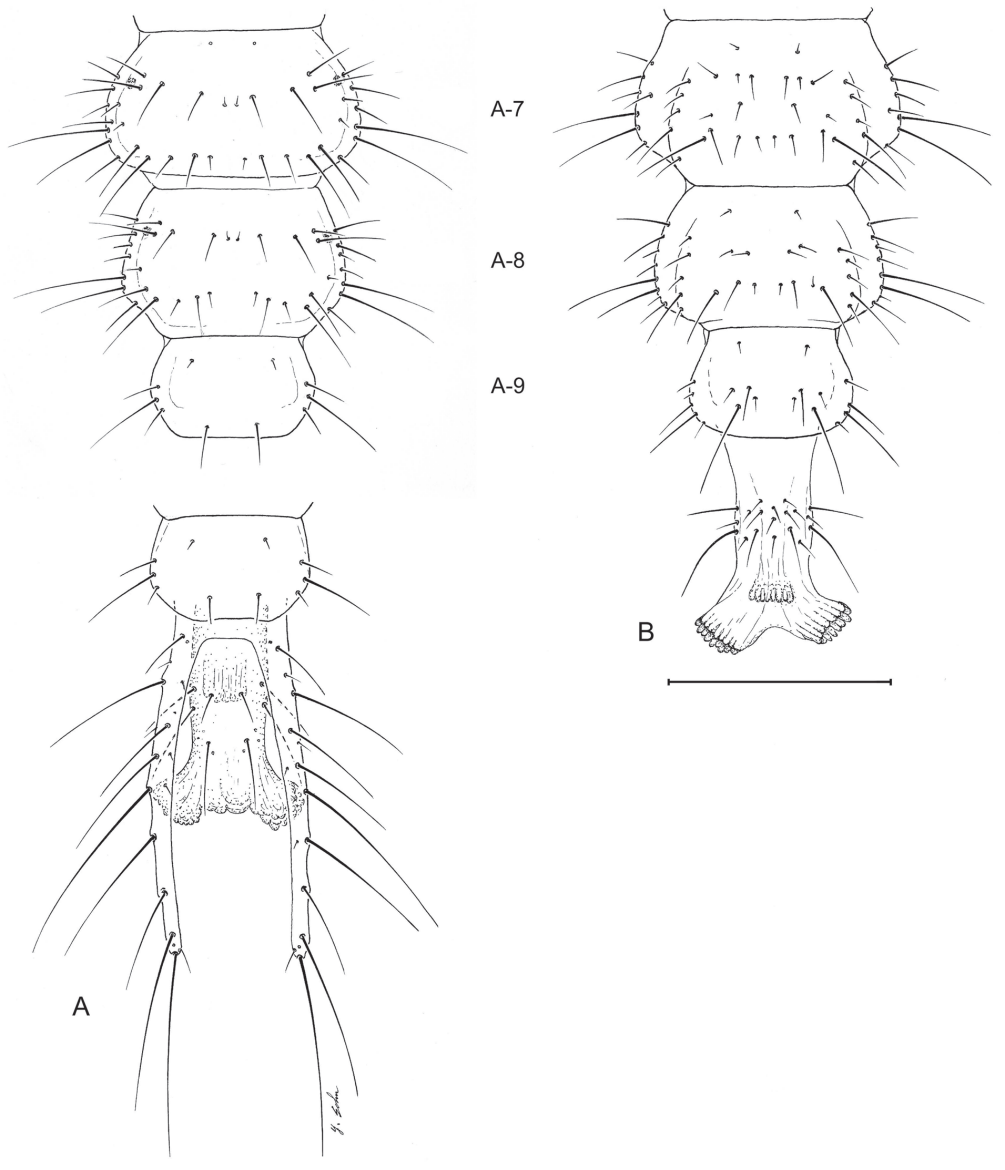
Larval abdomen (L_3_), of *Leptotrachelus dorsalis* (Fabricius). **A** dorsal aspect **B** ventral aspect. Abdominal segments **A-7** through **A-10** and cerci and pygidium. Scale line equals 0.5 mm.

##### Legs.

**Figure 12.**
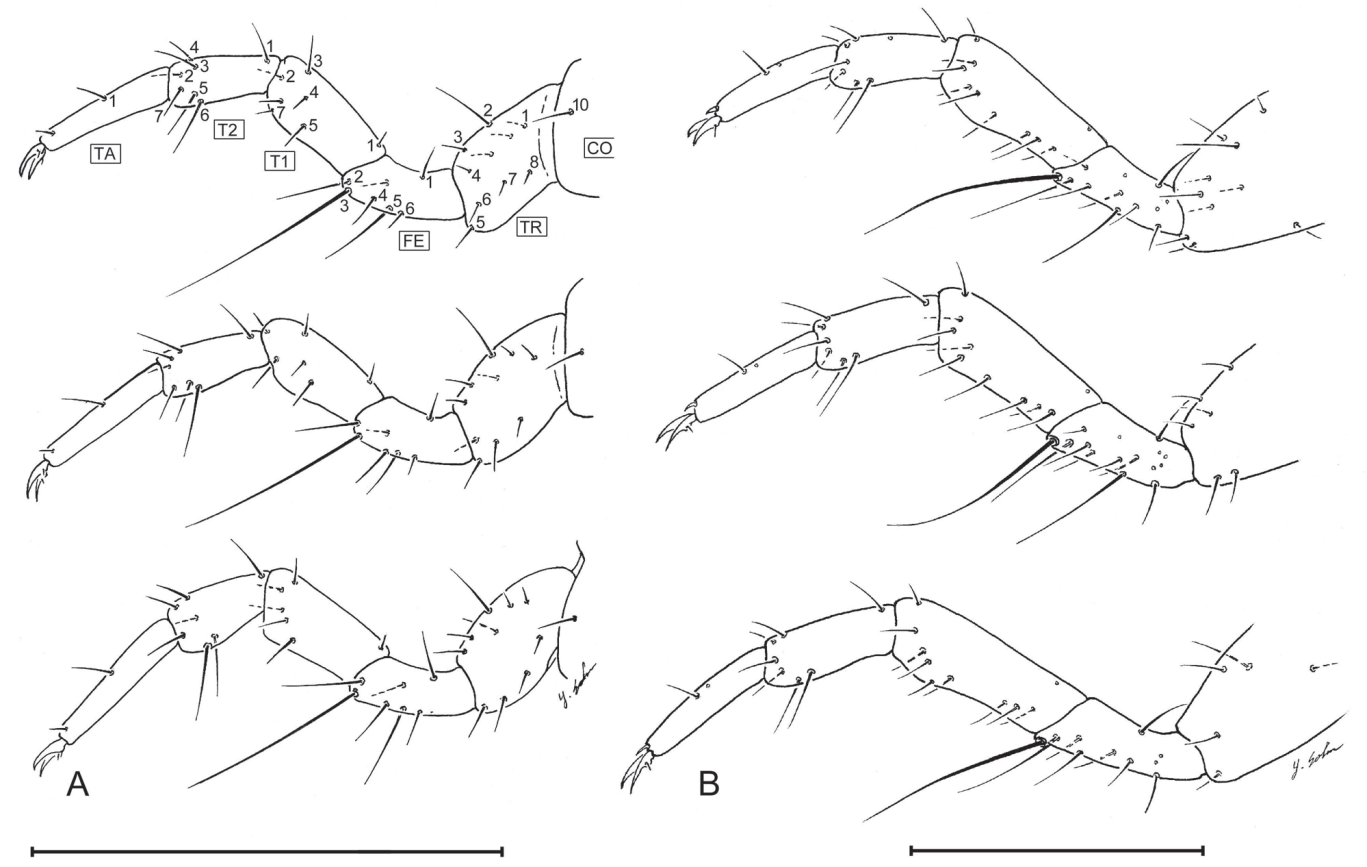
Coxa **CO** trochanter **TR** femur **FE** and tarsus **T1, T2, TA** of *Leptotrachelus dorsalis* (Fabricius), posterior lateral aspect. **A** L_1_, top **–** anterior leg; middle **–** middle leg; bottom **–** posterior leg **B** L_3_, top **–** anterior leg; middle **–** middle leg; bottom **–** posterior leg. Scale line equals 0.5 mm.

Coxa (Fig. 12) with 1 setae (CO10). Trochanter (Fig. 12) with 8 setae (TR1 **–** TR8), 2 unnumbered accessory setae and no pores. Femur (Fig. 12) with 6 setae (FE1 **–** FE6), 1unnumbered accessory seta and no pores. Tibia (Fig. 12) with 6 setae (TI1 **–** TI7, TI6 missing) and no pores. Tarsus (Fig. 12) 2 segmented, with 6 seta (T21 **–** T26) and 1 unnumbered accessory seta and no pores on T2, and 1 constant seta on T1 and 1 unnumbered accessory seta and no pores. Claws (Fig. 12) with 1 seta near base.

##### Description of pupa.

**Figure 13.**
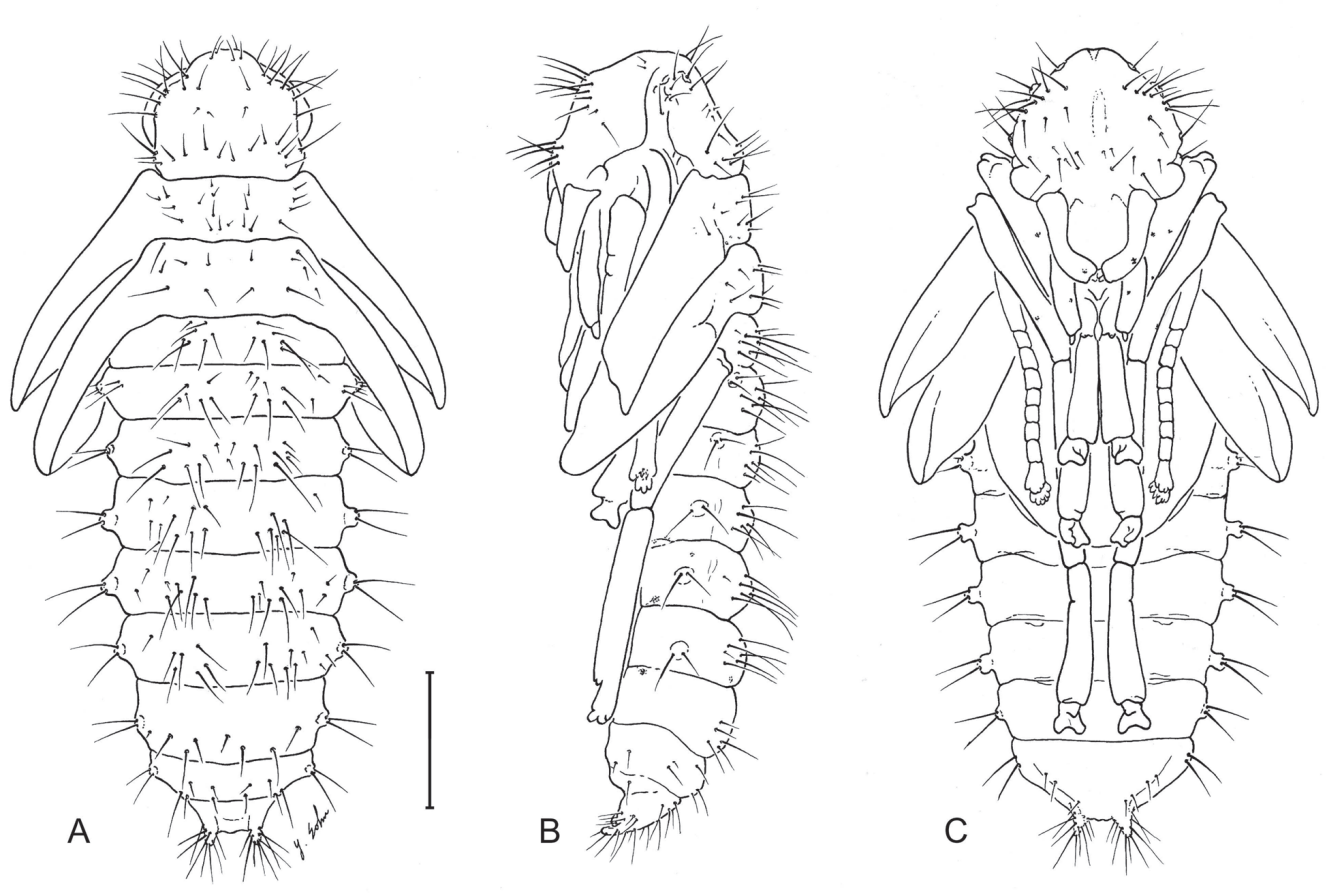
Pupa of *Leptotrachelus dorsalis* (Fabricius). **A** dorsal aspect **B** left lateral aspect **C** ventral aspect. Scale line equals 1.0 mm.

See Fig. 13. Typical of known carabid pupae, not many of which have been illustrated and described. Note the exceedingly setiferous ocular area of head and cerci.

**Figure 14.**
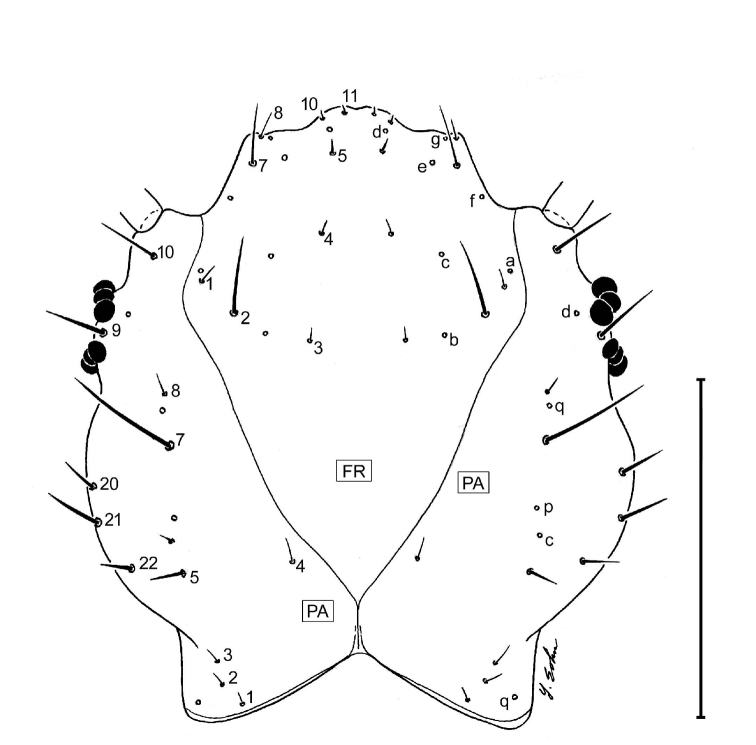
Larval head capsule (L_3_), parietale (PA), frontale (FR), dorsal aspect of *Askalaphium depressum* (Bates). This illustration was inadvertently left out of Erwin and Medina (2003); see references therein. Scale line equals 0.5 mm.

##### Characteristics of Ctenodactylini larvae.

Erwin and Medina (2003) showed that van Emden (1948) mixed attributes of two unrelated tribes, Odacanthini (Colliurini) and his concept of Ctenodactylini. They found that L_3_ members of the genus *Leptotrachelus* contain the following larval attributes found in larvae of *Askalaphium depressum*: epicranial suture short; cervical groove and keel present; maxilla with inner lobe present; neck not severely constricted; urogomphi nodal, yet not segmented. They located the collection of *Leptotrachelus dorsalis* larvae that van Emden studied (collected at Oxford, Indiana) in the NMNH, however, they are not in very good condition. They also confirmed his observations and added that second and third instars lack a pencillus and the terebral blade is micro serrate, but refrained from making a more detailed comparison until better specimens were discovered. These observations are now testable with the wealth of material found by the junior author in Houma, Louisiana. Our understanding of the structural attributes of the larval stages for the carabid tribe Ctenodactylini is now progressing.

Erwin and Medina (2003) also studied some poorly preserved specimens of *Odacanta melanura* L. in the NMNH collection. These specimens differ from ctenodactyline larvae in that the mandible has a single seta pensillus and the maxilla lacks an inner lobe, other features are not discernible. Undescribed larvae of some genera of ctenodactylines were also found in the rotten stems and leaf axils of species of the plant genera *Heliconia* L. and *Calathea* G. Mey in low wet places in Amazonian Ecuador and Perú. Adults of *Ctenodactyla* Dejean occur on species of these plants at night. Further discovery is necessary and subsequent documentation is required to define more accurately the Ctenodactylini, and to explore the patterns that must link these beetles evolutionarily and ecologically to the plants on which they live and the food which they eat.

## Conclusions

The hypothesized “home reed,” *Typha latifolia* L., as a microhabitat for this commensal species of carabids is classified in the Poales, Typhaceae. This reed, commonly called bulrushes or cattails, is an obligate wetland species and has been found in a variety of climates, including tropical, subtropical, southern and northern temperate, humid coastal, and dry continental up to 2300 m altitude in North, Middle, and South America. However, we point out that species of *Leptotrachelus* are known to occur commonly as adults on the culms of marsh grasses such as *Panicum dichotomiflorum* (*Leptotrachelus dorsalis*: Steiner 1984) in Maryland, USA, and *Paspalum* sp. (*Leptotrachelus* spp.: Erwin 1991) in the western Amazon Basin. There are currently 40 described species of *Leptotrachelus* in the Western Hemisphere, all with proximity to the spread of sugarcane plantations. In these plantations, there are often water-filled ditches with bulrushes and this is the likely source of the beetles that invade the standing canes; it is also a refuge for the beetles when the sugarcane fields are harvested in the fall and winter and resulting residues are burned off, a usual practice. They maintain their populations there in the bulrushes and reinvade the sugarcane ratoons in the spring, or after the next planting cycle. This makes them excellent biocontrol agents; however, broad spectrum pyrethroid insecticides sometimes used by farmers and predation by the red imported fire ant, *Solenopsis invicta* Buren, can severely reduce beetle numbers.

## Supplementary Material

XML Treatment for Leptotrachelus dorsalis
